# PD-L1 expression on immune cells is a favorable prognostic factor for vulvar squamous cell carcinoma patients

**DOI:** 10.18632/oncotarget.20911

**Published:** 2017-09-15

**Authors:** Jacek J. Sznurkowski, Anton Żawrocki, Katarzyna Sznurkowska, Rafał Pęksa, Wojciech Biernat

**Affiliations:** ^1^ Department of Oncological Surgery, The Medical University, Gdańsk, Poland; ^2^ Department of Pathology, The Medical University, Gdańsk, Poland; ^3^ Department of Pediatrics, Pediatric Gastroenterology, Hepathology and Nutrition, The Medical University, Gdańsk, Poland

**Keywords:** vulvar SCC, PD-L1, HPV, p16, prognosis

## Abstract

**Background:**

Anti-immune programmed death-ligand 1 (PD-L1) pathway is used by the tumor to overcome immune system and serves as immunotherapy target in various malignancies.

**Aim:**

To investigate the expression of PD-L1 in vulvar squamous cell carcinoma (vSCC) and to assess it's clinicopathological and prognostic significance.

**Methods:**

Immunohistochemical PD-L1 expression was evaluated in 84 vSCCs with previously defined status of p16 and DNA-HPV, infiltration of immune cells: CD8+, CD4+, FOXP3+, CD56+, CD68+, and GZB+ cells. PD-L1 positivity was defined as ≥5% of PD-L1-positive cells. Survival analyses included the Kaplan–Meier method, log-rank test and Cox proportional hazards model.

**Results:**

PD-L1 expression was detected on cancer and peritumoral immune cells. PD-L1-positivity of cancer nests (27/84, 32.1%) was correlated with higher infiltration of CD4+ (p=0.037), CD8+ (p=0.02), FOXP3+ (p=0.007), CD68+ (p=0.021) cells, while PD-L1 positivity of peritumoral immune cells (51/84, 60.7%) was correlated with higher infiltration of intraepithelial FOXP3+ cells only (p=0.037).

PD-L1-positivity of cancer cells but not immune cells, was more frequently observed in p16-negative tumors (p=0.004). High-risk HPV-status did not correlate with the PD-L1 status of cancer and immune cells (p=1.000) and (p=1.000) respectively). Median follow up was 89.20 months (range 1.7-189.5). PD-L1 positivity of peritumoral immune cells was found to be an independent favorable prognostic factor for OS. Conclusion: This study highlights the importance of comprehensive PD-L1 assessment in both cancer and immune cells. PD-L1 expression on peritumoral immune cells seems to be an additional prognostic factor in vSCC patients and may influence the results by anti-PD-L1 treatment.

## INTRODUCTION

The immune microenvironment of vulvar squamous cell carcinoma (vSCC) has been studied intensively by our group [1–3] and the others [4]. Lack of prognostic significance of adaptive immune effectors and regulatory T cells described in these studies has indirectly suggested limited role for immunotherapy for vulvar cancer patients.

Recently, we have found that cancer immune surveillance as represented by tumor infiltrating lymphocytes (TILs) and tumor associated macrophages (TAMs) depends on p16^INK4a^ expression regardless to high-risk HPV-DNA status [5] as various immune effectors contribute to improved clinical outcomes in patients with p16-positive and p16^INK4a^-negative tumors.

This suggests that not HPV infection itself, but p16^INK4a^ overexpression, contributes to shaping of the tumor microenvironment and p16^INK4a^-status could stratify patients for separate immunotherapeutic approaches in vSCC.

Recently, there has been a breakthrough in cancer immunotherapy against various cancer types by employing immune checkpoint blockade, particularly using antibodies directed against programmed death-ligand 1 (PD-L1) pathway members [6].

PD-L1 (also called B7-H1 or CD274), which is expressed on many cancer and immune cells, plays an important part in blocking the ‘cancer immunity cycle’ [7] by binding programmed death-1 (PD-1) and B7.1 (CD80), both of which are negative regulators of T-lymphocyte activation [6].

This study aimed to evaluate PD-L1 expression in vSCC tumors and to look for the correlation of this biomarker with clinical and pathological features of vSCC patients including TILs, TAMs as well as p16^INK4a^ and high risk (hr) DNA-HPV status in the primary tumor.

## RESULTS

### Patterns of PD-L1 expression

**Figure 1:**
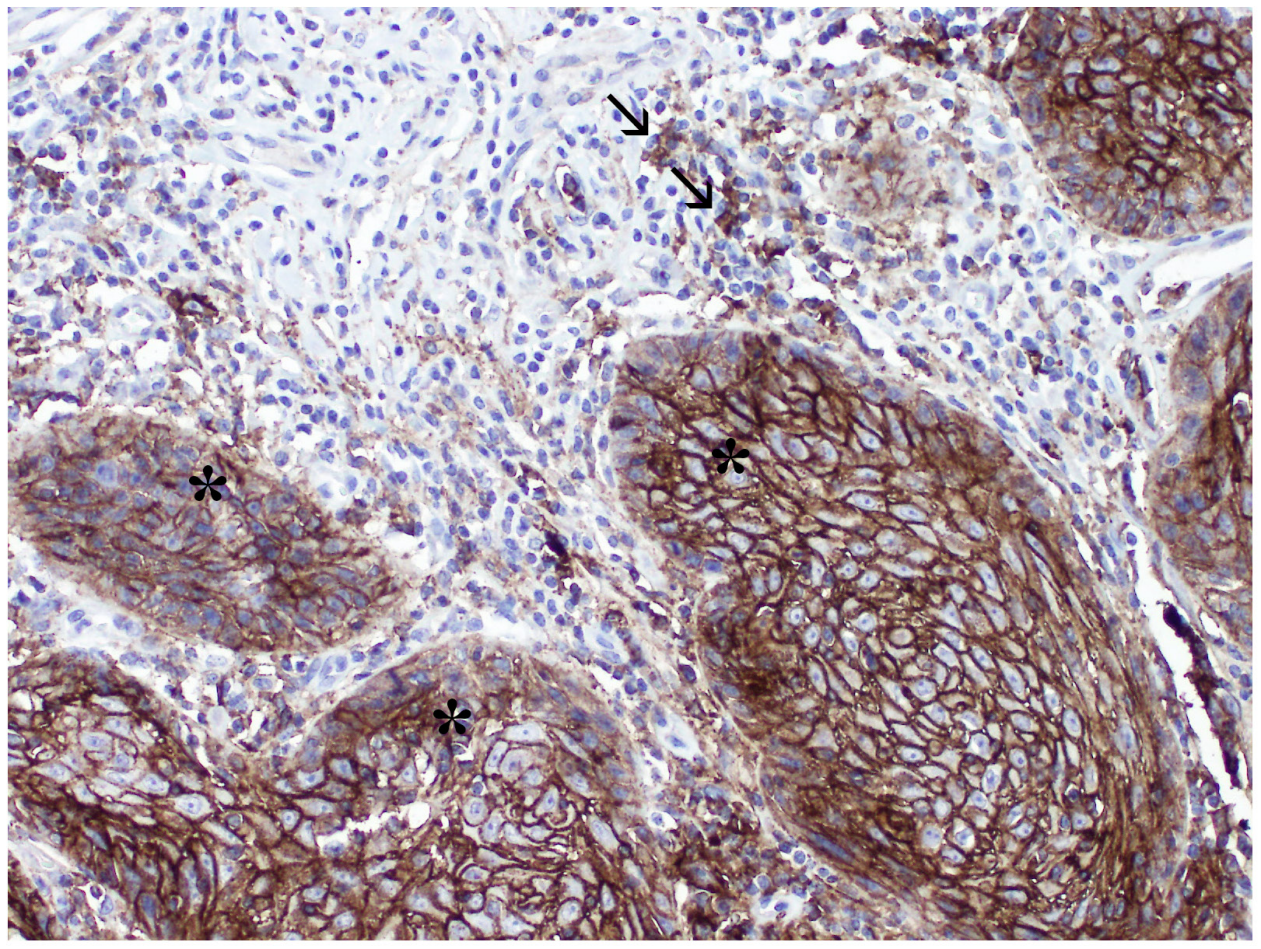
Microphotograph of immunohistochemical staining for PD-L1 within primary vSCC: (stars) expression on cancer cells, (arrows) expression on immune cells

PD-L1 expression was detected on cancer cells (CC-stars) and tumor-infiltrating immune cells (IC-arrows) (Figure 1).

In 27 of 84 tumors (32.1%) PD-L1 positive CC were found while PD-L1 positivity in peritumoral immune cells was disclosed in 51 of 84 (60.7%) cases.

**Figure 2:**
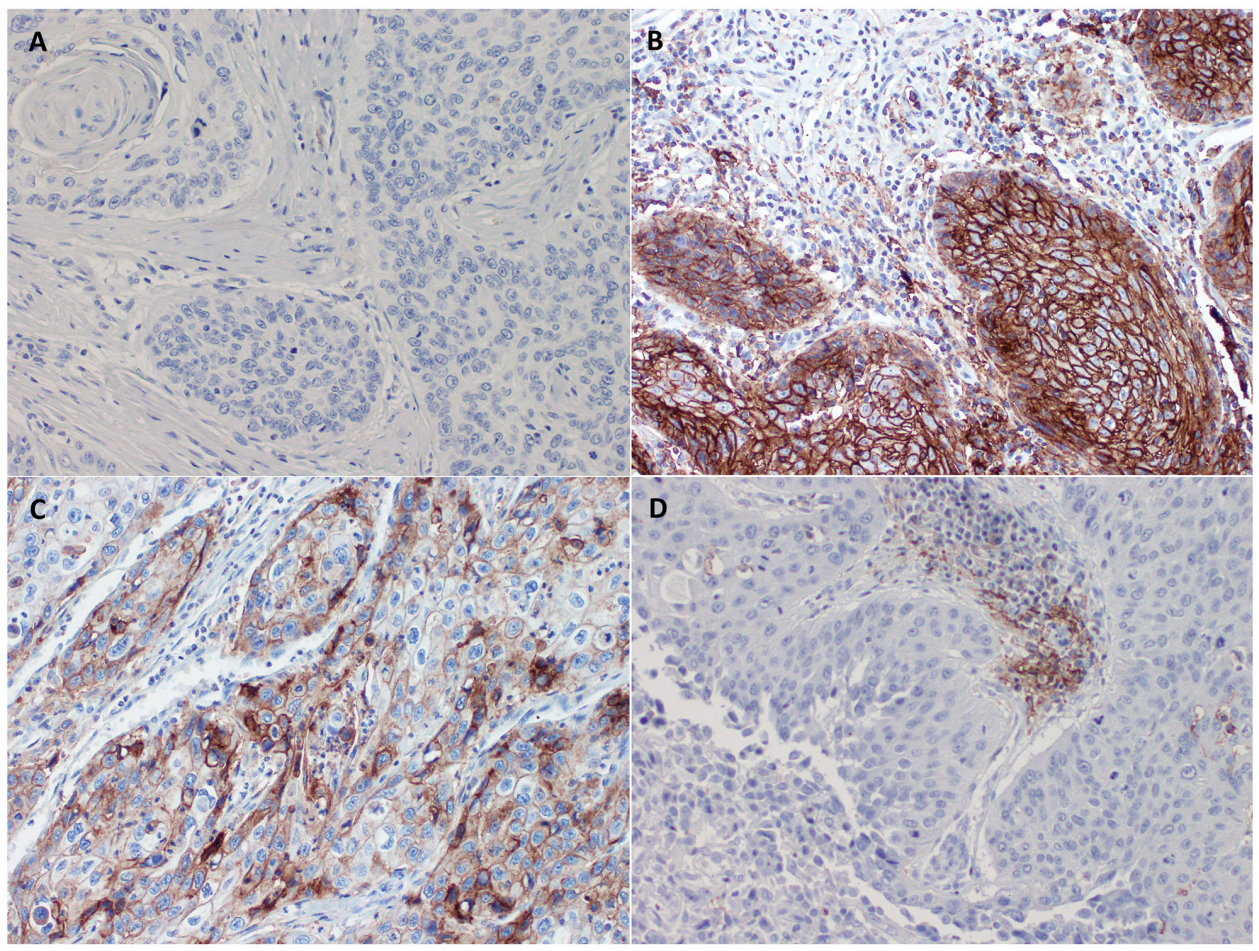
Microphotograph of immunohistochemical staining for CC-PD-L1/IC-PD-L1 variants within primary vSCC: **(A)** CC-PD-L1(+)/IC-PD-L1(+); **(B)** CC-PD-L1(-)/IC-PD-L1(-); (C) CC-PD-L1(+)/IC-PD-L1(-); (D) CC-PD-L1/IC-PD-L1(+).

Lack of PD-L1 expression (CC-PD-L1 and IC-PD-L1 negative tumors) was observed in 26 of 84 (31%) vSCC. Dual expression of PD-L1 (CC-PD-L1 and IC-PD-L1 positive tumors) was notified in 20 of 84 (23.8%) cases. PD-L1 was exclusively expressed on cancer (CC-PD-L1 positive and IC-PD-L1 negative) and immune cells (CC-PD-L1 negative and IC-PD-L1 positive) in 7/84 (8.3%) and 31/84 (36.9%) case respectively. Detected variants of PD-L1 expression are depicted on Figure 2.

### Association between PD-L1 expression and intraepithelial immune infiltrates

CC-PD-L1-positivity was correlated with higher intraepithelial infiltration of CD4+ (p=0.037), CD8+ (p=0.02), FOXP3+ (p=0.007), CD68+ (p=0.021) immune cells (Table 1).

**Table 1:** Correlation between CC-PD-L1 expression and subtypes of intraepithelial (IE) tumor infiltrating immune cells

	TC-PD-L1(pos.)	TC-PD-L1(neg.)	pUMW	IC-PD-L1(pos.)	IC-PD-L1(neg.)	pUMW
**CD 4+** median (range)	4 (0-21.66)	0 (0-18.33)	**0.037**	3 (0-21.66)	0 (0-19.66)	0.061
**CD 8+** median (range)	30.66 (0-214.33)	12.33 (0-121)	**0.002**	16 (0-214.33)	18.66 (0-101)	0.591
**FOXP3**+ median (range)	17.5(4.16-66)	11.33 (0-58.4)	**0.007**	17.8 (0-66)	9.28 (0-35.33)	**0.002**
**CD56+** median (range)	2(0-12.66)	2 (0-37)	0.520	2 (0-37)	1.66 (0-29)	0.163
**GZB+** median (range)	3.33(0-13.33)	2.83 (0-14.33)	0.303	3.33 (0-14.33)	3 (0-10)	0.183
**CD68+** median (range)	11.33(0-20.33)	7.33 (0-19.66)	**0.021**	8.5 (0-20.33)	7.66 (0-19.66)	0.607

IC-PD-L1-positivity of vSCC was correlated only with higher intraepithelial infiltration of FOXP3+ cells (p=0.037) (Table 1).

### Association of PD-L1 expression with p16 and high risk (hr) HPV statuses

CC-PD-L1-positivity was frequently observed in p16-negative vSCCs (p=0.004).

CC-PD-L1-positivity was similar in (hr)HPV-DNA positive and negative vSCCs (p=1.000).

Lack of difference in IC-PD-L1–positivity was observed in cases with mutually excluding p16 and (hr)HPV-DNA status (p=1.00 and p=1.00, respectively).

### Association of PD-L1 expression with common clinicopathological features

CC-PD-L1 and IC-PD-L1-positivity did not depend on pT (Fisher's exact test) (p=0.748 and p=0.158), presence of lymph node metastases (p=0.640 and p=0.656), tumor differentiation grade G1/G2,3 (p=1 and p=0.815), (Mann-Whitney U test) age (p=0.523 and p=0.072) and depth of invasion (p=0.361 and 0.502) respectively (Table 2).

**Table 2:** Association between PD-L1-positivity and clinicopathological features of vSCC patients

	CC-PD-L1(pos.)	CC-PD-L1(neg.)	p	IC -PD-L1(pos.)	IC -PD-L1(neg.)	p
**pT 1/2/3**	24/3/0	51/5/1	p=0.748	48/3/0	27/5/1	p=0.158
**Meta +/-**	14/13	25/32	p=0.640	25/26	14/19	p=0.656
**Grade 1/2+3**	9/18	18/39	p=1.000	17/34	10/23	p=0.815
**Age**	68 (36-82)	68 (40-85)	p=0.523	66 (36-85)	71 (44-85)	p=0.072
**Depth of invasion**	7.0 (2.0-18.0)	7.6 (2.0-16.0)	p=0.361	7.0 (2.0-18.0)	7.5 (2.0-13.0)	p=0.502

### Survival analyses

#### Entire cohort

**Figure 3:**
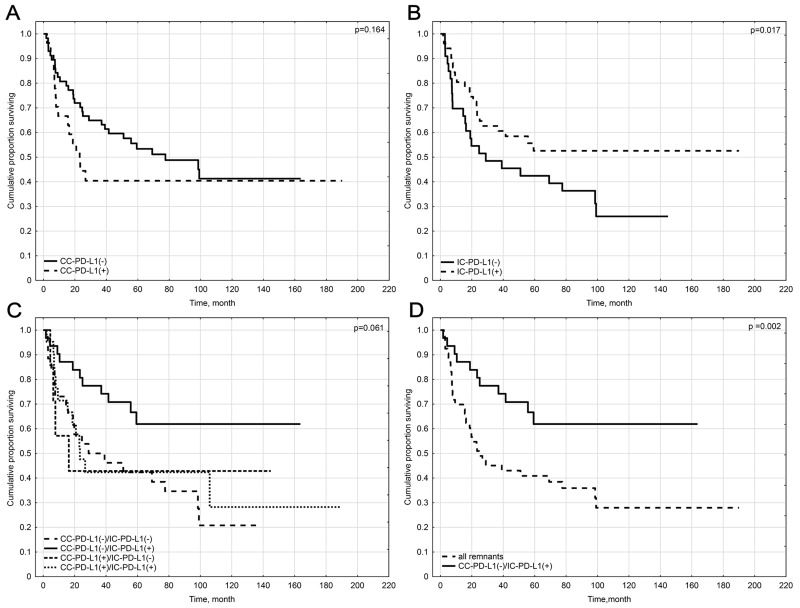
Kaplan-Meier survival curves for overall survival of patients by: **(A)** PD-L1-positivity of cancer nests in general population. **(B)** PD-L1-positivity of peritumoral immune cells in general population. **(C)** All Variants of CC-PD-L1 and IC-PD-L1 positivity in general population. **(D)** IC-PD-L1 positivity versus remaining variant of CC-PD-L1 and IC-PD-L1 positivity.

CC-PD-L1-positivity did not influence prognosis of vSCC patients (p=0.164) (Figure 3A).

Patients with primary tumors positive for IC-PD-L1 expression had improved OS compared to IC-PD-L1 negative ones (p= 0.017) (Figure 3B).

#### Variants of PD-L1 positivity

In the next step, the cohort of 84 vSCC patients was divided into four groups based on variants of CC-PD-L1 and IC-PD-L1-positivity: CC-PD-L1(+)/IC-PD-L1(-) n=7; CC-PD-L1(+)/IC-PD-L1 (+) n= 20; CC-PD-L1(-)/IC-PD-L1(+) n=31 and CC-PD-L1(-)/IC-PD-L1(-) n=26.

Survival analyses comparing all variants of PD-L1 expression indicated the group of patients with CC-PD-L1(-)/IC-PD-L1(+) tumors presented trend towards significantly better prognosis (p=0.061) (Figure 3C). When this group was further compared to the rest of cohort taken together it revealed significantly best outcome (p=0.002) (Figure 3D).

#### P16^INK4a^ status

Additionally, impact of PD-L1-positivity on survival was assessed in groups of patients with different p16^INK4a^ status.

CC-PD-L1-positivity did not influence the overall survival in cases having tumors p16^INK4a^-negative and p16^INK4a^-positive (p=0.3763 and p=0.1639 respectively).

**Figure 4:**
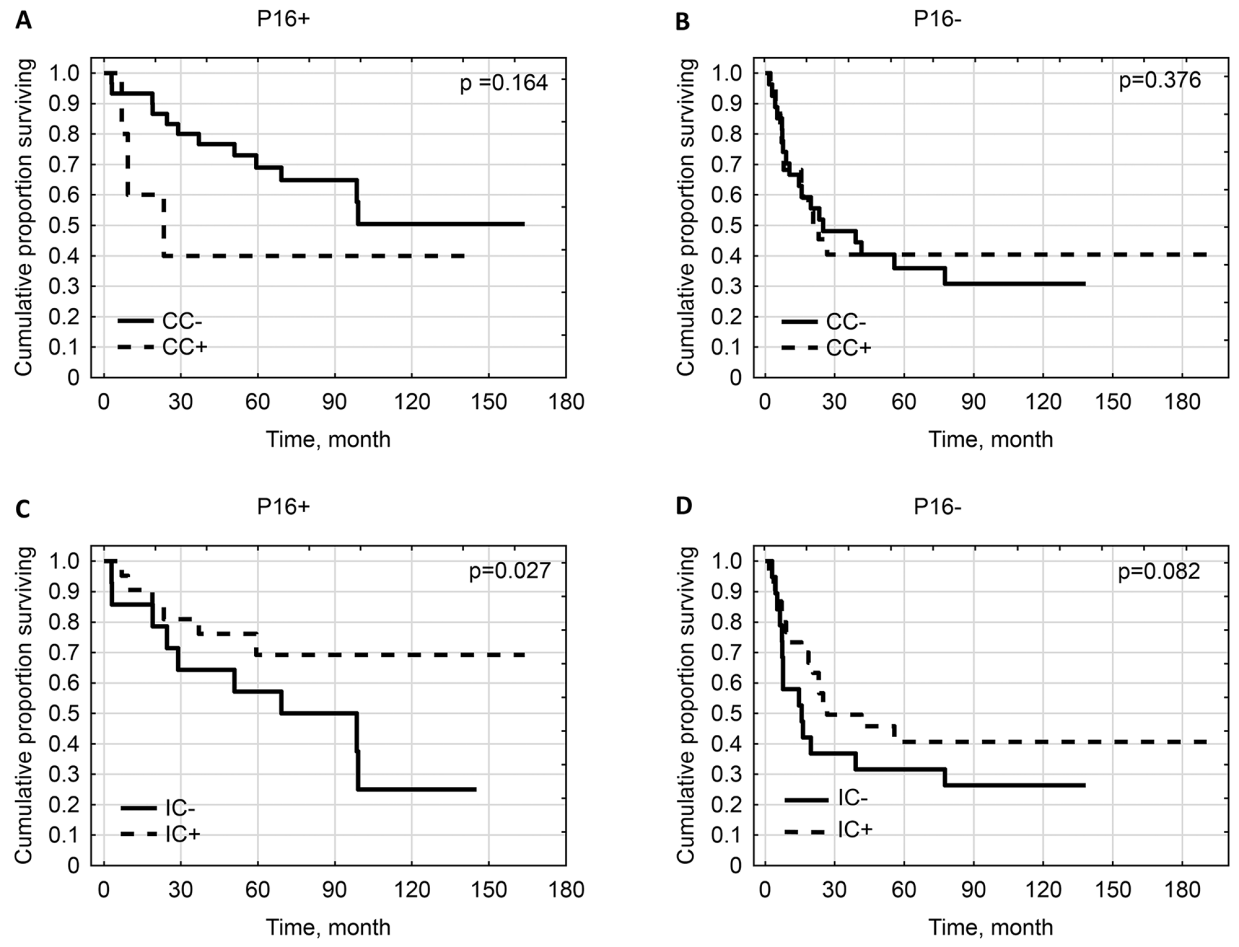
Kaplan-Meier survival curves for overall survival of patients by: **(A)** CC-PD-L1-positivity in population of patients with p16-positive tumors. **(B)** CC-PD-L1-positivity in population of patients with p16-negative tumors. **(C)** IC-PD-L1-positivity in population of patients with p16-positive tumors. **(D)** IC-PD-L1-positivity in population of patients with p16-negative tumors.

IC-PD-L1-positivity improved overall survival in cases with p16^INK4a^-positive tumors (p=0.0271) while it was not prognostic for patients with p16^INK4a^- negative tumors (p=0.0821) (Figure 4).

### Univarialble and multivariable analyses

Univariate and multivariate analyses revealed that IC-PD-L1-positivity was an independent prognostic factor for OS in vSCC patients (Tables 3 and 4).

**Table 3:** Univariate analyses of survival in vulvar cancer patients

Variables	Categories	Overall survival		p
		HR	95% CI	
Nodal status	metastases (-)	1	1.59-5.22	0.0005
	metastases (+)	2.88		
Adjuvant RTX	Yes	1	2.22-7.30	0.000005
	No	4.02		
Histologic grade	Low (G1)	1	1.65-7.15	0.001
	High (G2+G3)	3.43		
p16 status	Positive	1	1.11-3.81	0.0216
	Negative	2.06		
FIGO stage	I,II,III,IV	1.62	1.23-2.12	0.000592
Depth of invasion	Continuous	1.05	0.96-1.15	0.283261
Age	<60	1	1.12-5.59	0.025436
	>60	2.5		
IC-PD-L1	negative	1	0.32-1.03	0.0065
	positive	0.57		

**Table 4:** Multivariate analyses of survival in vulvar cancer patients

Variables	Categories	Overall survival		p
		HR	95% CI	
Nodal status	Negative for metastases	1	1.50-5.02	0.019
	Positive for metastases	2.74		
Histologic Grade	Low (G1)	1	1.33-5.90	0.007
	High (G2+G3)	2.80		
p16 status	Positive	1	1.13-3.95	0.001
	Negative	2.11		
IC-PD-L1	negative	1	0.25-0.83	0.010
	positive	0.45		

## DISCUSSION

In the present study, we comprehensively evaluated PD-L1 expression on the whole sections of tumor tissue showing that this biomarker is present both on cancer and immune cells. In our opinion assessment of macrodissected cancer tissue is superior than evaluation of tumor microarrays (TMAs). Compared with the whole tumor sections, tumor microarrays (TMAs) may be less representative, especially while assessing the biomarkers expressed in the tumor infiltrating immune cells [8].

PD-L1 positivity was defined as ≥5% of cells regardless the intensity of membranous staining. This is consistent with several publications analyzing this biomarker within other malignancies [9–12]. An appropriate cutoff value in validating the positive expression of PD-L1 remains contentious. Subgroup analysis with different cutoff values has shown that there was a contradictory trend when using the cutoff value of `5%’ or `1%’ in evaluating the correlations of PD-L1 positive expression with survival of cancer patients [8]. Therefore, a combined classification of cutoff values for PD-L1 assessment in CCs and ICs seems feasible and reasonable.

PD-L1-positivity of immune cells was more frequently observed within vSCC than PD-L1-positivity of cancer cells (60.7% vs. 32.1%). We found only two publications dealing with PD-L1 in vulvar cancer [13, 14]. None of these papers provided data neither on patterns of PD-L1 staining within primary tumor nor on frequency of PD-L1 expression in vSCC.

Recently we have reported that p16^INK4a^-overexpression modulates immune cells infiltration regardless to (hr)HPV-DNA status [5]. Patients with p16^INK4a^-negative tumors although more infiltrated with TILs (CD8+, CD4+, GZB+ cells) had worse outcome than cases with p16^INK4a^-positive vSCCs [5, 15]. Here CC-PD-L1 expression was frequently observed on more infiltrated p16^INK4a^-negative vSCCs suggesting that anti-immune programmed death-ligand 1 (PD-L1) pathway is at least partially responsible for worse outcome in these patients. However, CC-PD-L1 did not reveal prognostic significance either in entire cohort nor in patients with p16^INK4a^-negative tumors.

The mechanism by which CC-PD-L1 surface expression is induced is quite ambiguous and includes either immune cell and cancer cells. Besides adaptive PD-L1 up-regulation in an inflammatory cytokine milieu caused by TAMs and/or Interferon-γ [16–18], cancers can have innate potential to drive PD-L1 expression by oncogene. Several genetic alterations were associated with constitutive PD-L1 up-regulation, like PTEN loss or NPM/ALK [19, 20]. Amplification at 9p24.1, where PD-L1 resides, has been associated with PD-L1 up-regulation in oral squamous cell carcinoma [21] and non-small cell lung cancer [22]. Activation of PI3K pathway by PTEN loss in both breast cancers [23] and glioblastomas [19] has been shown to induce PD-L1 expression. These events have long been known to result in the expression of neoantigens, differentiation antigens, or cancer testis antigens, which can lead to presentation of peptides bound to major histocompatibility class I molecules on the surface of cancer cells, distinguishing them from their normal counterparts [7]. This could partially explain, observed in this study, correlation between CC-PD-L1-postivity and higher intraepithelial immune infiltration as represented by CD4+, CD8+, FOXP3+ T cells. Additionally, CC-PD-L1-positivity was more frequently observed among p16^Ink4a^-negative tumors which were found to be more infiltrated by TILs and TAMs [5]. Lack of prognostic effect of interaction between PD-L1 on cancer cells and PD-1 on TILs can reflect state of equilibrium between host immune response and cancer tissue, thus not influencing patient's overall survival.

Interestingly, PD-L1-positivity of immune cells was found to be independent prognostic factor for overall survival of vSCC patients. Similar findings were published for head and neck cancer patients suggesting that comprehensive assessment of PD-L1 expression could bring an information for more precise usage check point inhibitors [9]. Recent meta-analysis of prognostic value of PD-L1 expression on tumor infiltrating immune cells, including 3674 patients with different types of cancers, confirmed that IC-PD-L1 is related to a better survival of cancer patients [8].

Several previous reports have shown that PD-L1 expression is a negative prognostic factor in several cancer types, including renal, colorectal, and lung cancers [24–26], but others have reported that PD-L1 is a favorable prognostic factor in metastatic melanomas, NSCLC, and Merkel cell carcinomas [27–29]. In the majority of these studies, the expression of PD-L1 in tumor sections has been evaluated without exact discrimination between IC and TC.

Notably, we have found that IC-PD-L1-positivity improves survival in entire cohort, but when the patients were subdivided into two groups based on p16^INK4a^-status of the primary tumor, this protective effect was observed only for p16^INK4a^-positive cases. (hr)HPV-DNA status did not influenced PD-L1-positivity either on cancer and immune cells which supports our previous results suggesting lack of clinicopathological significance of HPV-DNA positivity within vSCC [5, 15].

When we separately analyzed PD-L1-positivity on CC and IC, which was categorized into four variants of vSCC cases, we found that patients with CC-PD-L1(-)/IC-PD-L1(+) tumors had significantly best outcome.

Few hypotheses could explain this paradoxical favorable prognosis of PD-L1 expression on ICs. IC-PD-L1 expression could be driven by adaptive mechanisms such as exogenous inflammation mediated immune attack and then reflected pre-existing immunity [9, 30]. IC-PD-L1-positivity could have stronger relations with cancer immune response, and probably depends on tumor microenvironments. Such favorable profile of immune microenvironments was described for PD-L1 expression in pulmonary squamous cell carcinoma [12].

Here, IC-PD-L1-positivity was correlated with unfavorable intraepithelial FOXP3+ lymphocytes while CC-PD-L1-positivity was correlated with favorable CD4+, CD8+ as well as unfavorable FOXP3+and CD68+ immune cells. Thus, indication of more favorable pre-existing immunity remains unclear.

However, with the exception of PD-L1 expression all other data were retrieved from our previous studies, originality of current report is supported by completely new study project and design. Immunohistochemical PD-L1 expression has never been analyzed before within vulvar cancer tissue. By analyzing all available data for this cohort, we were able to present in single publication, not only patterns of expression and prognostic significance of this biomarker but also associations with TILs, TAMs, p16^INK4a^ and (hr)HPV-DNA status. Such correlations have never been described before.

This study provides a new insight into immune surveillance on vSCC as for the first time demonstrates that although PD-L1 could mediate the occurrence of cancer immune escape, it also indicates an effective immune response.

The weaknesses of the current study are the retrospective design and the small size of cohort involved. Its strength lies in the consistency of the treatment of patients under uniform standards and their long observation, revealing recurrences and hence enabling the assessment of the prognostic significance of all the analyzed biomarkers.

## MATERIALS AND METHODS

This retrospective study was approved by the Polish Ministry of Science and Higher Education review board, who determined that further informed consent was not required as informed consent for tissue sampling was obtained from all patients prior to surgical treatment and written consent was given by the patients for their information to be stored in the hospital database and used for research.

### Population

Our cohort of 85 patients previously studied with known p16^INK4a^ and (hr)HPV status[15], as well as TILs and TAMs [5] was included into further analyses. One case was excluded due to lack of tissue for PD-L1 staining. Briefly, the median age of the 84 patients was 68 years (range 36-85), the median duration of follow-up was 88.75 months (range 1.7-189.5), The 5-year disease free survival (DFS) rate was 61.75 %.

### Tissue samples

We analyzed all 84 primary tumors for PD-L1 expression. Data on TILs were retrieved from our previous studies [1–3, 5]. Data on p16^INK4a^ and (hr)HPV status were retrieved from our recent study [15].

### Antibodies

Mouse anti-human monoclonal antibody against PD-L1 (clone 22C3, cat. No. M3653) was obtained from Dako Inc. Mouse anti-human monoclonal antibodies against CD4 (NCL-L-368), CD8 (NCL-L-295), CD56 (NCL-CD56-1B), were obtained from Novocastra, Inc. Mouse anti-human monoclonal antibody against FOXP3 (cat. No ab20034) and CD68 (cat. No ab955) were obtained from Abcam, Inc. Mouse anti-human polyclonal antibody against Granzyme B (cat. No 760-4283) was obtained from Ventana Medical Systems, Inc. Mouse anti-human monoclonal antibody against p16 (cat. No sc-56330) was obtained from Santa Cruz Biotechnology Inc., USA [5].

### Immunohistochemistry

Immunohistochemical staining for PD-L1 was performed as follows. Four-micron-thick serial sections were cut, placed onto slides, and deparaffinized. For epitope retrieval, slides were immersed in Target Retrieval Solution (pH 6.1; Dako Cytomation, Denmark) and heated in a pressure cooker. Then, they were incubated for 90 min with primary antibody (1:50 dilution, clone 22C3, cat. nr. M3653). The reaction was visualized using the EnVision FLEX (DAKO). Appropriate positive (tonsil) and negative (primary antibody replaced with normal mouse IgG at an appropriate dilution) controls were included in each staining. Immunohistochemistry results were evaluated by two independent pathologists blind to the clinical data.

PD-L1 cell expression was referred as positive if membranous staining was identified and showed a continuous honeycomb pattern. Cytoplasmic staining of PD-L1 was disregarded. Expression of PD-L1was categorized into two groups according to the percentage of PD-L1-positive cells. PD-L1 positivity was defined as ≥5% of cells regardless to intensity of membranous staining [9–12].

Immunohistochemical staining for other antibodies: p16, CD8, CD4, FOXP3, CD68, CD56, GZB with evaluation and classification of TILs and p16^INK4a^ status was previously described for this cohort by our group [5].

### Detection of high risk HPV-DNA

Tissue dissection and DNA preparation as well as mucosal HPV DNA amplification and genotyping were detailed for this cohort in our other recent study [15].

### Statistical analysis

The statistical analysis was performed using the Fisher's exact probability test. The difference between numerical variables was verified by Mann-Whitney U test.

Overall survival curves were estimated by the Kaplan–Meier method and compared by the two-sided Fox test. Univariate and multivariate analysis was performed using Cox regression model. *p* values <0.05 were regarded as significant in all analyses.

All analyses were performed using the statistical software

Statistical 13 (Stat Soft Inc.).

## CONCLUSION

PD-L1 expression on IC, not on CC, is independent predictor of favorable OS in surgically treated vSCC patients. Assessment of the expression of immune-related molecules in whole tissue section slides could produce evidence relevant to the appropriateness of treatment via immune checkpoint blockade.

Moreover, our findings highlight the importance of comprehensive assessment of both TC and IC in vSCC and suggest that usage of antibodies directed against PD-L1-pathway seems to be favorable for subjects having tumors negative for PD-L1 peritumoral immune cells. Thus, incorporating peritumoral immune cells into the classification of PD-L1 expression seems to be necessary to select the beneficial vSCC patients for anti-PD-L1 treatment.
